# Maximum Power Point Tracking for Cascaded PV-Converter Modules Using Two-Stage Particle Swarm Optimization

**DOI:** 10.1038/s41598-017-08009-7

**Published:** 2017-08-24

**Authors:** Mingxuan Mao, Qichang Duan, Li Zhang, Hao Chen, Bei Hu, Pan Duan

**Affiliations:** 1 Automation College, Chongqing University, Chongqing, 400044 China; 2 School of Electronic and Electrical Engineering, University of Leeds, Leeds, LS2 9JT United Kingdom; 3 Center for energy and environment policy, Beijing institute of technology, Beijing, 100081 China; 4 State Grid Chongqing Electric Power Company Nan’an Power Supply Subsidiary Company, Chongqing, China; 5 Judge Business School, University of Cambridge, Cambridge, CB2 1AG United Kingdom

## Abstract

The paper presents a novel two-stage particle swarm optimization (PSO) for the maximum power point tracking (MPPT) control of a PV system consisting of cascaded PV-converter modules, under partial shading conditions (PSCs). In this scheme, the grouping method of the shuffled frog leaping algorithm (SFLA) is incorporated with the basic PSO algorithm, ensuring fast and accurate searching of the global extremum. An adaptive speed factor is also introduced to improve its convergence speed. A PWM algorithm enabling permuted switching of the PV sources is applied. The method enables this PV system to achieve the maximum power generation for any number of PV and converter modules. Simulation studies of the proposed MPPT scheme are performed on a system having two chained PV buck-converter modules and a dc-ac H-bridge connected at its terminals for supplying an AC load. The results show that this type of PV system allows each module to achieve the maximum power generation according its illumination level without affecting the others, and the proposed new control method gives significantly higher power output compared with the conventional P&O and PSO methods.

## Introduction

**Figure 1:**
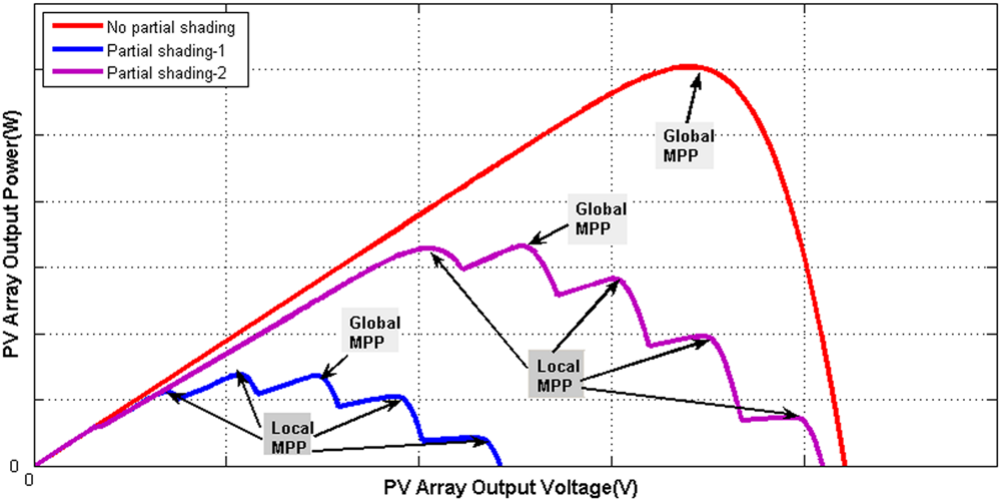
Examples of the P-V characteristics curves of a PV array composed of series-connects PV modules for the different irradiations.

Solar Photovoltaic (PV) electricity generation has been actively explored throughout the world and its use has grown ever more rapidly over the last decade. PV power generators are becoming a familiar landscape, ranging from small (less than 5 kW) residential PV panels, larger (hundreds of kW) building integrated installations, and PV farms up to the megawatt range in open areas. However there are still problems impairing its performance, particularly the output power reduction due to partial shading. Since each of the PV arrays is composed of multiple PV modules connected in a chain, each module may be exposed under different light levels (e.g., due to moving clouds, shading from surrounding buildings, trees or poles, and building integrated PV applications, etc.), then the output characteristics of the PV array exhibits multiple local maximum power points (Local MPPs) shown in Fig. 1, resulting in the energy loss. This issue occurs commonly in urban domestic PV systems. In addition grid compliancy, reliability and service lifetime are also issues attracting attention. Active research and development work has continued, focusing on two aspects: the topologies and configurations for the PV systems, and the control schemes for maximum power extraction. Regarding the former, the traditional structure has series connected DC strings of PV modules with a bypass diode around each module for protection in the event of partial shading. Many such strings may be connected in parallel to a central dc-ac inverter when grid connection is required. The main shortcoming of this is its inability to control the modules according to variable and different illumination levels, resulting in lower power generation, as the series connection forces an equal terminal current throughout a string. A new approach considers the use of small power converters for each PV module or a group of them. These can be low cost non-isolated dc-dc^1, 2^ or dc-ac converters^3, 4^, and the unit formed by a converter and its associated PV panels may be termed an integrated PV-converter module. When such modules are connected in a series chain, the converters, assuming they are controllable, permit a variable ratio between the PV cell current and the series chain current. Comparing with the traditional structure this configuration can be controlled at the individual module level and hence can be more efficient in coping with the variability of environmental and climate conditions, and especially the partial shading. The correct set of converter ratios allows every integrated module to work at its individual maximum power point (MPP).

Regarding the control aspects, various optimization schemes have been developed to achieve the maximum power point tracking (MPPT) aiming to extract as much power as the PV panel can generate irrespective the illumination conditions, though most of these were developed for PV panels with uniform characteristics. For example the well-known perturb and observe (P&O) method is simple, and requiring minimal knowledge for MPP searching however it causes constant disturbances to the system and is slow to converge. Other methods include the artificial intelligence schemes, such as fuzzy logic controller (FLC)^5, 6^ and artificial neural network (ANN)^7, 8^. Alternative approaches use evolutionary algorithms such as particle swarm optimization (PSO) algorithm^9, 10^, artificial fish swarm algorithm (AFSA)^11^, artificial bee colony (ABC) algorithm^12^ or shuffled frog leaping algorithm (SFLA)^13^. The common feature of these methods is that they all rely on a PV model of some forms hence a prior test or information about the PV system being controlled is required. Once a sufficiently accurate model is obtained they converge fast without perturb the system. However none of these methods alone, including P&O, can work well with PV panels consisting of modules of varied I-V characteristics. The power-voltage characteristics feature multiple power peaks which require more advanced searching schemes to find the global maxima. For the PV system configured by chained integrated PV-converter modules, a robust control scheme is required to combine with the searching algorithms to extract the maximum power available from individual modules.

With the above stated issues this paper presents a new control scheme for a PV system comprising a chain of integrated PV step-down dc-dc converter modules. According to the number of cascaded modules the system dc-voltage has multiple levels and may be converted to ac via a dc-ac converter. The control scheme adopted from the PSO algorithm and combined with the SFLA can search for multiple peaks for each PV module. Furthermore for the switch control of dc-dc converters of PV modules it uses a permutation Pulse-Width Modulation (PWM) scheme allowing switching sequence swapping for balanced switch utilisation. This achieves the overall system MPPT irrespective the pattern of illumination.

The rest of the paper is organized as follows: In Section 2, the PV system configuration and operation principle is described. The new maximum power point tracking scheme based on the TSPSO algorithm is presented in detail in Section 3. In addition, The system control scheme is described in Section 4. Section 5 presents and discusses the experimental results. Finally, we give the conclusion in Section 6.

## PV System Configuration and Operation Principle

**Figure 2:**
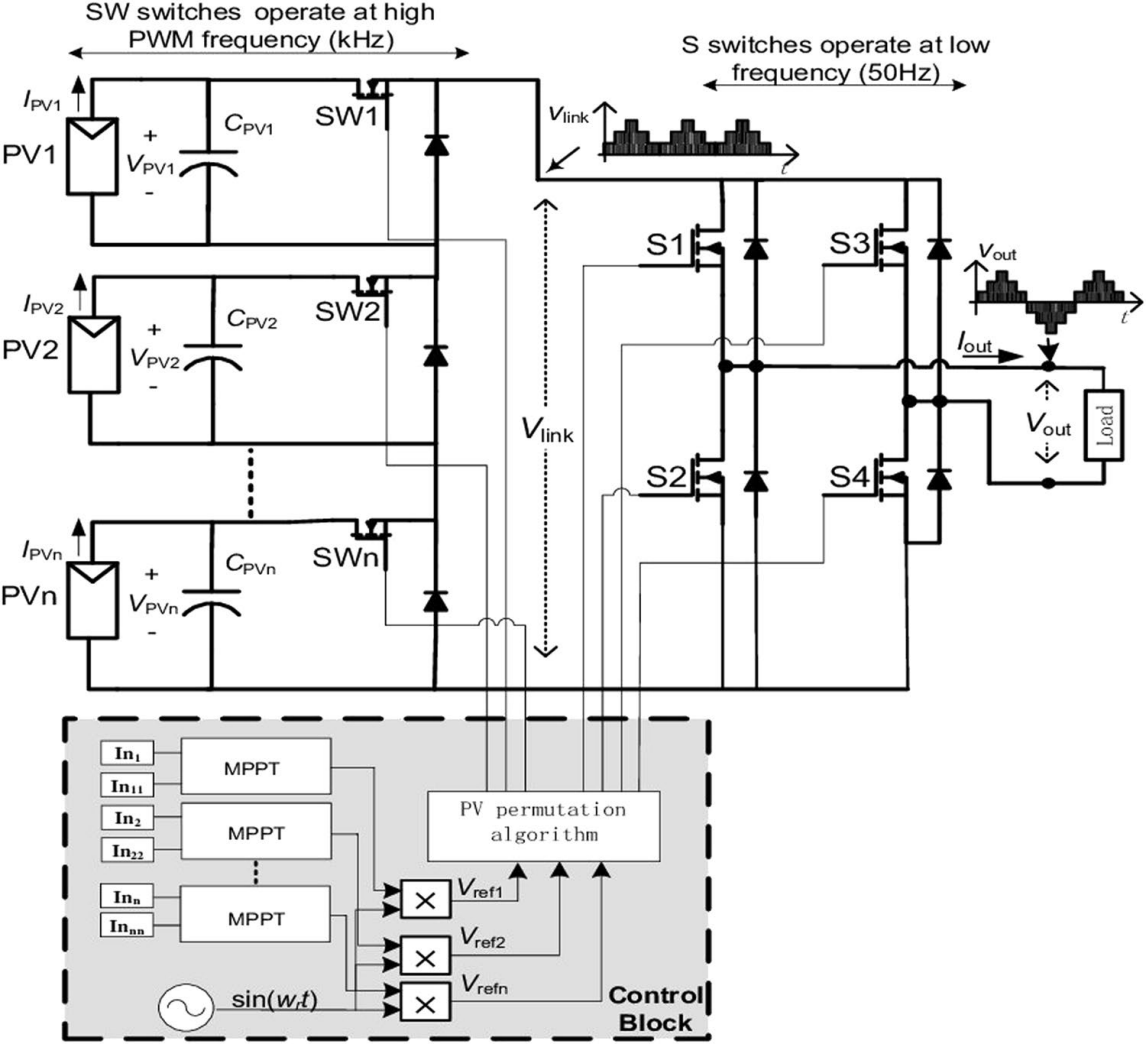
PV system with multilevel DC- link converter and the proposed MPPT method.

The configuration of the studied PV system could comprise *n* cascaded integrated PV-converter modules as shown in Fig. 2, though in this paper for ease of discussion only two modules are considered. This originally proposed structure^14^ can generate multiple dc voltage levels. However in this work multiple levels of ac output voltage are produced by multiplying each dc level voltage with a unity-amplitude sinewave of the required output frequency. The resultant waveform is fed to a dc-ac H-bridge controlled using a square wave rather than any sine-triangle PWM schemes. This simply changes the sign of the output to produces the negative segments of the cycle, hence enabling the output to supply an AC load or an AC grid. In this example the system output is connected to a restive load *R*_*L*_ plus a filter inductor *L*. With an appropriate MPPT scheme, each integrated PV-converter module is able to control its PV panel terminal voltage to reach the peak power point, and hence the system can obtain the maximize power generation irrespective of the variability of illumination level on each PV modules.

## Proposed Maximum Power Point Tracking Scheme

This MPPT scheme needs to work well for PV modules operating under all illumination conditions including partial shading. As is well-known for the partially shaded PV panels/modules their P-V characteristics present multiple peaks. The challenge to any MPPT scheme is able to find the global peak accurately and rapidly.

The proposed new scheme combines the well-known particle swarm optimization (PSO) algorithm with a clustering method, the shuffled frog leaping algorithm (SFLA). The algorithm is implemented in a two-stage procedure, hence is named a Two-Stage PSO (TSPSO). With the PV system of multiple chained PV converter modules as shown in Fig. 2, this TSPSO algorithm is applied to each of the *n* modules simultaneously to track their respective MPPs.

### Principles of PSO Algorithm

PSO^15^ is an evolutionary computation technique proposed by Kennedy and Eberhart in 1995. Originated from observing the behavior of bird flocks searching food in an area, PSO adoptes the scenario by appling it to solve optimization problems. In PSO, a set of randomly placed particles is initialized; each particle represents a potential solution and has a corresponding fitness value derived from a fitness function. The objective is to find the optima by updating generations of particles. Assuming a space containing *S* particles, the updating velocity and position of the i^th^ particle at the k^th^ iteration are respectively denoted as *V*_*i*_^*k*^ and *X*_*i*_^*k*^. In the iterative process, the updated position of this particle at the (*k* + 1)^*th*^ time step is influenced by the information of its own best position *P*_*i*_ and the global best *P*_*g*_ at the *k* ^*th*^ step. The velocity and particle position update formulas are written as follows:

1 $${V}_{i}^{k+1}=\omega {V}_{i}^{k}+{c}_{1}{r}_{1}({P}_{i}^{k}-{X}_{i}^{k})+{c}_{2}{r}_{2}({P}_{g}^{k}-{X}_{i}^{k})$$

2 $${X}_{i}^{k+1}={X}_{i}^{k}+{V}_{i}^{k+1}$$

where *ω* is the inertia weight factor, *c*_1_ and *c*_2_ are the acceleration factors, *r*_1_, *r*_2_ are random values ∈(0, 1), and *k* is the iteration order. To prevent the particles searching blindly, their velocities and positions are limited to the ranges defined respectively by [*V*_*min*_, *V*_*max*_] and [*X*_*min*_, *X*_*max*_].

Applying the PSO algorithm to search the MPPs of a PV array the particles are the PV terminal voltages. The fitness value for each particle (voltage) is the output power of the PV array which is evaluated using a simplified form of original Bishop model^16^ defined as

3 $${I}_{out}={I}_{sc}-{I}_{o}[\exp (\frac{q{V}_{j}}{AK{T}_{c}})-1]-{I}_{shunt}$$

4 $$Fitness\,Function=Power({{\rm{G}},{\rm{T}},{\rm{I}}}_{out})={I}_{c}\times {V}_{out}$$

where *I*_*sc*_ is the photo current, the second current on the RHS formula is due to P-N junction leakage, *V*_*j*_ is the P-N junction voltage and *I*_*shunt*_ represents the PV panel ohmic leakage current. Definitions of other parameters in (3) are given in the Supplementary information.

The defect of the traditional PSO algorithm is that it cannot cope well for the maximum power point searching of a PV system under unequal and changing illumination conditions. With multiple optimal points fast and accurate searching for maxima, the algorithm requires a large population size to cover a wide area which is certain to contain all the optima. This makes it slow to converge.

### Two-Stage PSO Algorithm with Multiple Swarms

The proposed new PSO algorithm takes into account the specific feature of a PV generation system operating under PSCs, namely that the P-V characteristic exhibits multiple peaks. The number of these peaks depends on the number of chained modules and their respective illumination levels, and only one of them corresponds to the global maximum power point (MPP). Since the PSO algorithm can only deal with local search effectively, it is natural, in this application, to combine it with a scheme which can partition the searching space into multiple sectors corresponding to the number of modules in a PV array so that local parallel searching leading to the global solution can be performed. SFLA is considered ideal for performing this with PSO due to its special features. This is a meta-heuristic algorithm, and its working principle is summarized as follows. The algorithm works on a population of particles (frogs), each of which has a fitness value which measures its quality in terms of the required solution. By partitioning these particles into multiple subsets also called memeplexes, according to their ranking orders, the algorithm performs local searches for optimal solutions within each memeplex. The results obtained are then shuffled so that the information can be passed. This local search and shuffling process continues until a defined convergence criterion is met. Applying this to MPPT for a PV array under PSCs, we firstly use the SFLA concept to assess a population of particles; these are the voltage values covering the whole P-V curve of an array according to the fitness function given by eqs (3) and (4). These can then be partitioned into several groups and in this case the division can be made according to the number of modules in the array. The above two-stage procedure and the equations used are detailed as follow.

#### Stage (1)

All particles are divided into several groups according to the grouping idea of the SFLA, and in each group/swarm, with the local best already obtained, the speed and position of each particle within the group are updated by using the equations:

5 $${v}_{mn}^{k+1}=\omega {v}_{mn}^{k}+{c}_{1}{r}_{1}({P}_{m}^{k}-{x}_{mn}^{k})$$

6 $${x}_{mn}^{k+1}={x}_{mn}^{k}+{v}_{mn}^{k+1}$$

7 $$\omega ={\omega }_{\max }-({\omega }_{\max }-{\omega }_{\min })\frac{K}{J}$$

where *m* = 1, 2, …, *M* being the number of groups, *and n* = 1, 2, …, *N*, is the number of particles within a group. *P*_*m*_ is the best position of particles in the *m*_*th*_ group. Having obtained *n* particle positions at the (*k* + 1)*th* step, we evaluate their fitness values which are the power values and then acquire the best particle. Subsequently, a new local best position for the *m*_*th*_ group, *P*_*m*_, is derived. In the eq. (7), *K* is the generation index representing the current number of evolutionary generations. The maximal and minimal weights *ω*_*max*_ and *ω*_*min*_ have been set to 0.9 and 0.4 in this paper.

#### Stage (2)

Using *m* local best particles to find the one giving the maximum power amongst them, this is chosen as the optimal for the entire population at the *kth* iteration step, i.e the global best. The speed and position of the local best particles are updated by the formulas:

8 $${v}_{m}^{k+1}={c}_{2}{r}_{2}({P}_{{\rm{g}}}^{k}-{P}_{m}^{k})$$

9 $${P}_{m}^{k+1}={P}_{m}^{k}+{v}_{m}^{k+1}$$

where *P* ^*k*^_*g*_ is the best position of particles in the entire swarm at the *kth* iteration.

It is worth highlighting that this updating process is fast since the population size is determined by the number of groups and this is small. In the current application *m* = 3. In the iterative procedure, when the best value within a group is equal to the global best value, the (*P*_*g*_ − *P*_*m*_) in eq. (8) is zero, so the iterative process converges. The algorithm also stops when a predefined maximum iteration step count *J* is reached.

### Implementation Procedure of the Proposed Algorithm

**Figure 3:**
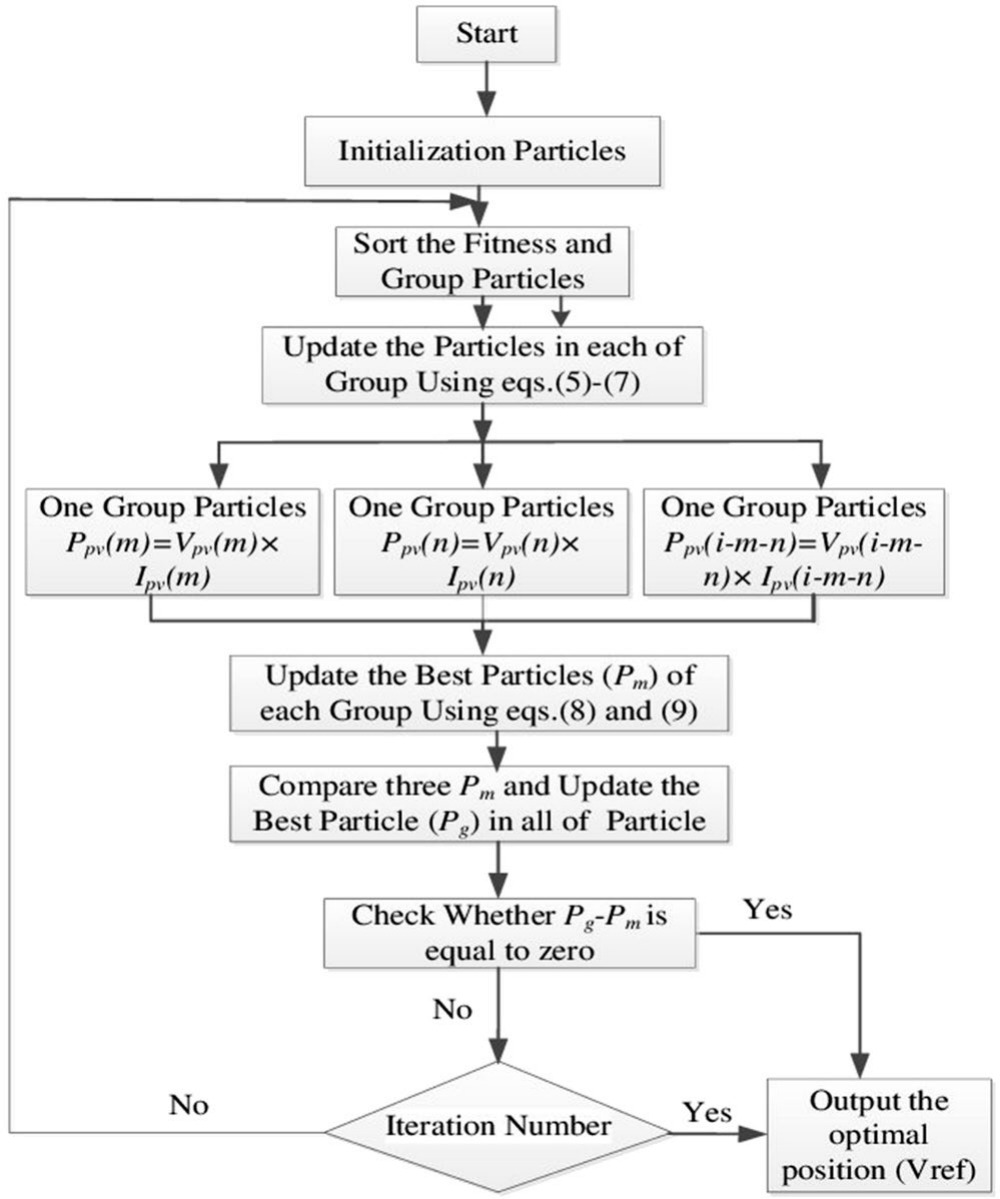
The complete flowchart of the proposed method.

This is shown in the flowchart in Fig. 3. Initially a set of particles is randomly chosen, these being voltage values within the output voltage range of the PV array, or duty ratios when a DC-DC converter is used. These are divided into groups according to the module number, and the maximum P_m_ in each group is determined by evaluating their respective fitness function values. Then the proposed two-stage PSO is applied to update the individual particle positions in each group using eqs (5)–(7), and the local maximum P_m_ using eqs (8) and (9). These result in m newly updated local peaks, and the one giving the highest power is set as the global peak at the kth iteration. The process is repeated until the iteration process converges.

## Control Scheme for Cascaded PV-Converter System

The control scheme for the PV system shown in Fig. 2 should ensure the terminal voltage of each integrated PV converter unit as close as possible to its individual reference level established by the above described TSPSO MPPT method. It consists of three steps^14^: (1) the direct PWM determining the output voltage levels and dc-dc converter duty ratios; (2) the switch permutation algorithm to determine the switching states for the PV-converter units for building up the output voltage of the system; (3) the dc-ac H-bridge converter is used to change the multilevel DC voltage waveform to a single-phase AC waveform of low frequency by tracking the sinusoidal reference signal.

For clarification, a graphical illustration of the process for generating the output waveform in the basic system with two modules is given in Table 1. Table 1 presents the switching states and output voltage generated for the system with two PV sources (*n* = 2), and gives a more practical demonstration of the generated output voltage through the permutation algorithm, and in each case the non-controlled PV source is used to form the basis voltage for the controlled PV source.

**Table 1:** Output Generation from Two PV Sources.

C	*v*_offset1_	*v*_offset2_	Output generation per switching period *T*_*s*_
1	0	0	
	1	0	
	0	1	
	1	1	
2	0	0	
	0	1	
	1	0	
	1	1	

## Simulation Results and Discussions

### Simulation Static Experiment

The simulation studies were performed on a system having two identical cascaded PV and step-down converter units, hence generating two dc-voltage levels, under different partial shading conditions. An H-bridge connected at the system output terminals is used to convert the dc to ac voltage of five-level and supplies a resistive load. The parameters of the PV source at panel surface temperature of 20 °C are listed in Table 2. The different partial shading patterns are divided into four cases listed as follow: Case 1, G_1_ = 350 W/m^2^, G_2_ = 500 W/m^2^; Case 2, G_1_ = 500 W/m^2^, G_2_ = 700 W/m^2^; Case 3, G_1_ = 1000 W/m^2^, G_2_ = 500 W/m^2^; Case 4, G_1_ = 1000 W/m^2^, G_2_ = 700 W/m^2^. For comparing the output voltage waveform performance, the Fast Fourier Transformation (FFT) is applied to analyze the harmonic components present in the waveform and the total harmonics distortion (THD) factors for all cases are evaluated.

**Table 2:** Parameters of the PV panels simulated.

Symbol	Parameter	Value
*P*_*mpp*_	Maximum power at 1 kW/m^2^	25 W
*V*_*oc*_	Open circuit voltage	19.76 V
*I*_*sc*_	Short circuit current	3.286 A
*C*_pv_	PV source terminal capacitor	2200 μF
*R*	Load resistance	10 Ω
*L*	Load filter inductance	5 mH
*f*	AC output frequency	50 Hz

To verify the effectiveness of the proposed new TSPSO MPP scheme under PSCs, the conventional P&O method and PSO algorithm are also applied to the cascaded five-level PV-converter system and the results are compared. The step voltage of the conventional P&O method used in the paper is set 0.1 V. Table 3 shows the basic parameters used in the PSO algorithm and the proposed algorithm, including the inertia weight *ω*, acceleration factors *c*_1_ and *c*_2_, number of particles *S*, number of groups *M*, and maximum iterative number *J*.

**Table 3:** The basic parameters of two algorithms.

Method	*c*_*1*_	*c*_*2*_	*w*	*S*	*M*	*J*
PSO	0.6	0.8	0.5	12	—	3
TSPSO	0.6	0.8	self-adaption (*ω*_*max*_ = 0.9, *ω*_*min*_ = 0.4)	12	3	3

**Figure 4:**
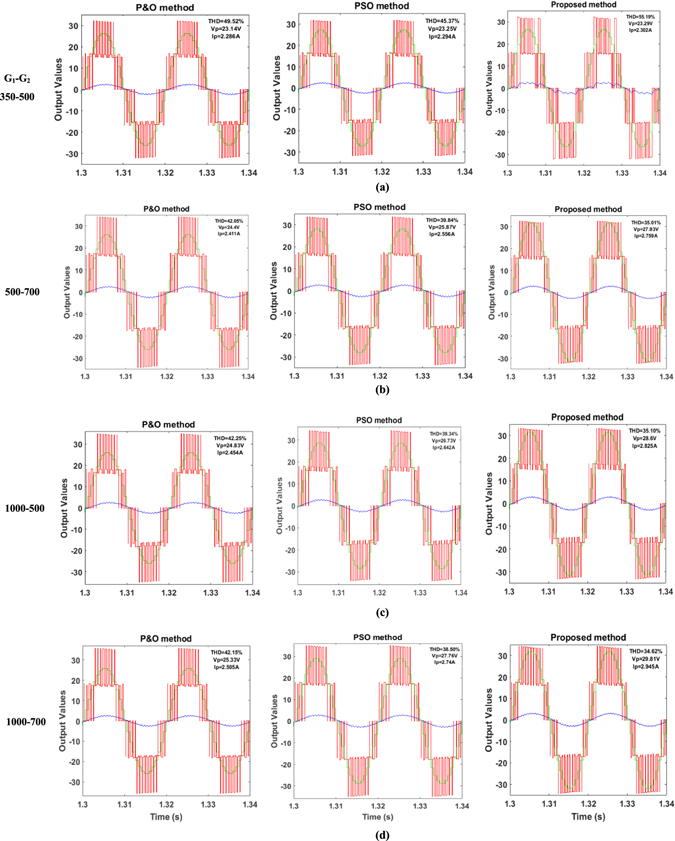
Output voltage (red line), output current (blue line) and reference voltage (green line) waveforms of five-level converter measured under the control by P&O method (column 1), the traditional PSO method (column 2) and the proposed algorithm (column 3).

**Figure 5:**
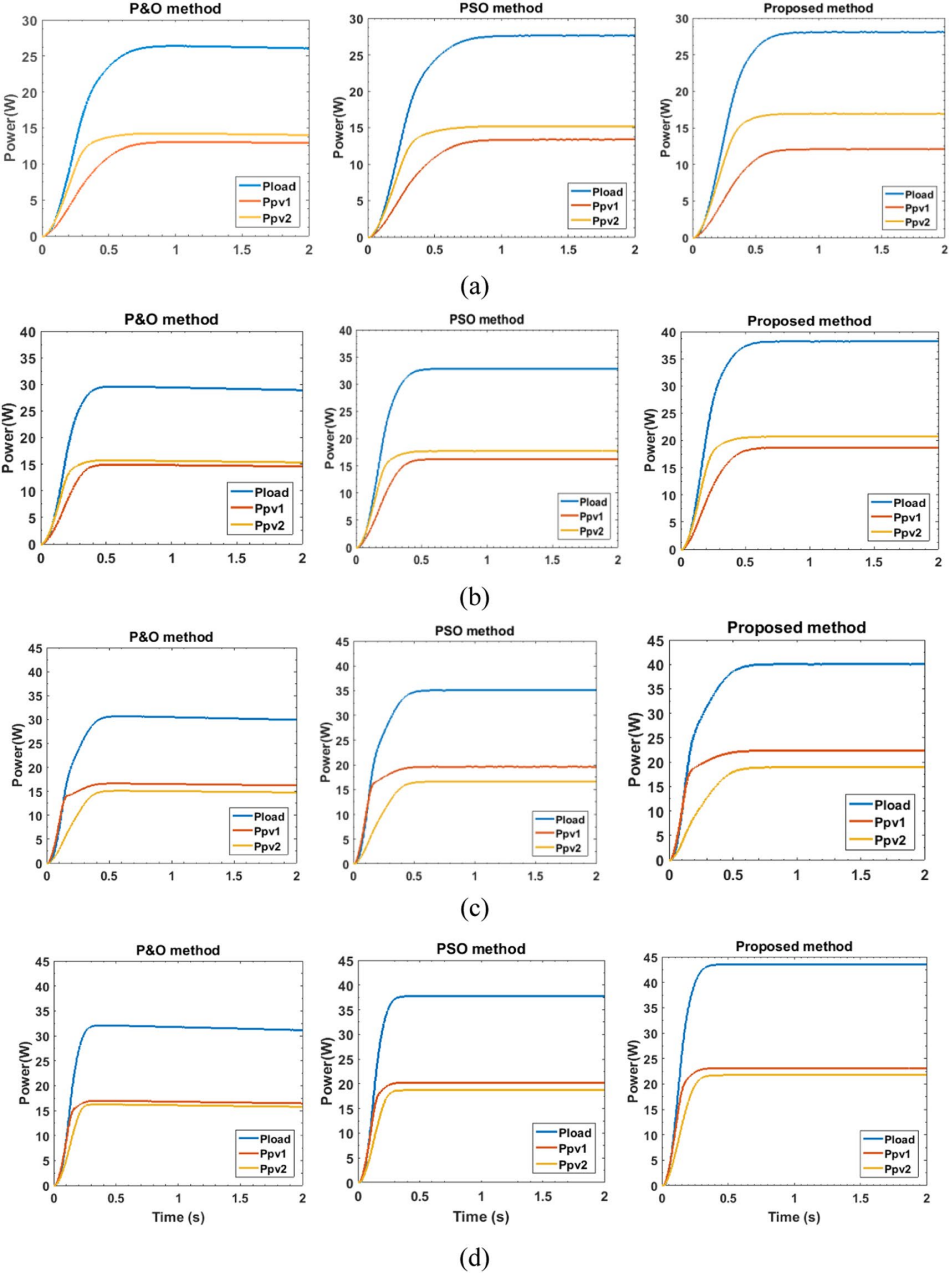
Load power (*P*_*load*_), pv1 power (*P*_*pv1*_) and pv2 power (*P*_*pv2*_) of five-level converter measured under the control by P&O method (column 1), PSO method (column 2) and the proposed method (columns 3).

Figure 4 shows the plots of the reference voltage, ac load voltage and current waveforms from respectively P&O, PSO and TSPSO methods under different illumination conditions. Figure 5 depicts the powers generated by PV-converters 1 and 2 and the total load received power corresponding to the conditions set in Fig. 4. To show clearly the performance differences between each method the numerical values for the power and THD values are also listed in Table 4. It is evident that the proposed TSPSO algorithm outperforms both P&O and conventional PSO methods. In all cases its total output power is higher than that obtained by the other two methods. In particular, comparing to P&O the total output power of TSPSO is always higher, the maximum difference between them is as high as 27.5%. In contrast PSO method can harvest more power than from P&O, but it is still less efficient than TSPSO in all cases studied. The power output of the later can be about 13% higher the maximum compared to the former. In terms of load voltage waveform performance assessed via THDs, as can be seen in Table 4, the proposed TSPSO gives persistently lower values than that of other two methods for all cases.

**Table 4:** Measured power values to the load and THD values under various shading conditions.

Case	P&O method				PSO method				Proposed method			
	P_pv1_	P_pv2_	P_Load_	THD (%)	P_pv1_	P_pv2_	P_Load_	THD (%)	P_pv1_	P_pv2_	P_Load_	THD (%)
1	13.02	14.17	26.29	49.52	13.36	15.18	27.62	45.37	12.09	16.93	28.08	55.19
2	14.74	15.51	29.24	42.05	16.2	17.70	32.83	39.84	18.33	20.69	36.92	35.01
3	16.41	14.93	30.29	42.25	19.62	16.60	35.09	39.34	22.34	18.37	39.17	35.10
4	16.67	15.96	31.54	42.15	20.22	18.72	37.73	38.50	23.11	20.73	42.15	34.62

### Simulation Dynamic Experiment

**Figure 6:**
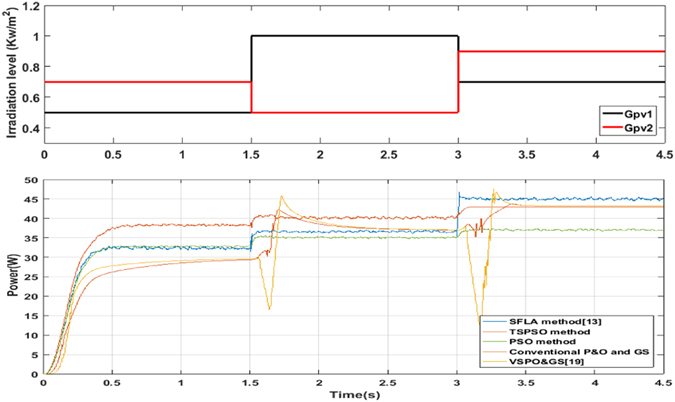
Output results of the three methods: the changing process of illumination levels of two PV sources, and output power curves from the five methods.

To vividly describe the advantage of the scheme with the novel MPPT algorithm, further tests are done on the PV system with the conventional P&O method with global scanning method (fixed step = 0.01), the PSO method, the SFLA method^13^, the VSPO&GS method^17^ and the proposed method under fast transient variations of shading patterns. The parameters of these methods are as mentioned above. The fast transient variations of shading patterns include: Starting from case 1 initially, the switch to case 2 occurs at t = 1.5 sec., and case 2 to case 3 at t = 3 sec. as shown in Fig. 6, where Gpv1 and Gpv2 respectively represent the irradiation levels of PV1 and PV2 under fast transient variations of shading patterns.

Figure 6 shows the variations of output load power (*P*_*load*_) of the PV system, using the five methods, under three fast transient variations of shading patterns. It can be clearly observed from Fig. 6 that although the output power of the proposed method has a little oscillation during the MPP searching processes, the power delivered to load of the proposed method obviously outperforms the other methods in case 1 and case 2. Concretely speaking, the output power delivered to the load of the proposed method is improved about 5 W on average compared to the SFLA method in case 1 and case 2. Also the proposed method reduces the oscillation effectively in case 3.

## Conclusion

In the paper, a new control scheme has been proposed, which includes the TSPSO based-MPPT algorithm and a PWM permutation algorithm. In the scheme, firstly, the shuffled frog leaping algorithm (SFLA) and adaptive speed factor is incorporated in the conventional PSO algorithm to ensure fast and accurate searching of the global extremum. This scheme has been applied to control a multilevel DC-Link converter with a PWM algorithm to deal with the problem of partial shading. The proposed control scheme was simulated using Matlab–Simulink for a multilevel DC-Link converter with five-level AC output voltages under different partial shading conditions. The results have been compared with those from P&O, and PSO methods. It has shown that the maximum output power harvested from the proposed method can be about 13% higher than the PSO method and 27.5% higher than the P&O. The performance of the output voltage waveform is also superior than that from the two conventional schemes, since it gives significantly lower THD values compared to those obtained by the later.

## Electronic supplementary material

Supplementary Information

## References

[1] Singh SN (2017). Selection of non-isolated DC-DC converters for solar photovoltaic system. Renewable and Sustainable Energy Reviews. Renew Sustain Energ Rev.

[2] Muhammad, M., Armstrong, M. & Elgendy, M. Analysis and Implementation of High Gain Non-isolated DC-DC Boost Converter. IET *Power Electr* (2017).

[3] Navarro, D., Vazquez, N. & Cortes, D. Programmable reference generator to control a DC/AC converter for solar applications. *Proceedings of the International Conference on Scientific Computing* (*CSC*) 234 (2015).

[4] Irfan MS, Ahmed A, Park JH (2017). Current-Sensorless Power-Decoupling Phase-Shift Dual-Half-Bridge Converter for DC–AC Power Conversion Systems Without Electrolytic Capacitor[J]. IEEE T Power Electr.

[5] Cheng PC, Peng BR, Liu YH, Cheng YS, Huang JW (2015). Optimization of a fuzzy-logic-control-based MPPT algorithm using the particle swarm optimization technique. Energies.

[6] Rezvani A, Gandomkar M (2016). Modeling and control of grid connected intelligent hybrid photovoltaic system using new hybrid fuzzy-neural method. Sol. Energy.

[7] Lin FJ, Lu KC, Ke TH (2016). Probabilistic Wavelet Fuzzy Neural Network based reactive power control for grid-connected three-phase PV system during grid faults. Renew. Energy.

[8] Messalti S, Harrag A, Loukriz A (2017). A new variable step size neural networks MPPT controller: Review, simulation and hardware implementation. Renew Sustain Energ Rev.

[9] Renaudineau H (2015). A PSO-based global MPPT technique for distributed PV power generation. IEEE Trans. Ind. Electron..

[10] Manickam C, Raman GR, Raman GP, Ganesan SI, Nagamani C (2016). A Hybrid Algorithm for Tracking of GMPP Based on P&O and PSO With Reduced Power Oscillation in String Inverters. IEEE Trans. Ind. Electron..

[11] Mao, M., Duan, Q., Yang, Z. *et al*. Modeling and global MPPT for PV system under partial shading conditions using modified artificial fish swarm algorithm. *IEEE International Symposium on Systems Engineering* (*ISSE*) 1–7 (2016).

[12] Sawant, P. T., Lbhattar, P. C. & Bhattar,C. L. Enhancement of PV system based on artificial bee colony algorithm under dynamic conditions. *IEEE International Conference on Recent Trends in Electronics*, *Information & Communication Technology* (*RTEICT*) 1251–1255 (2016).

[13] Sridhar R, Jeevananthan S, Dash SS (2017). A new maximum power tracking in PV system during partially shaded conditions based on shuffled frog leap algorithm. J Exp Theor Artif In.

[14] Abdalla I, Corda J, Zhang L (2016). Optimal control of a multilevel DC-link converter photovoltaic system for maximum power generation. Renew Energ.

[15] Eberhart, R. & Kennedy, J. A new optimizer using particle swarm theory. In *Proceedings of the Sixth International Symposium on Micro Machine and Human Science* 39–43 (1995).

[16] Bishop JW (1998). Computer Simulation of the Effects of Electrical Mismatches in Photovoltaic Cell Interconnection Circuits. Solar Cells.

[17] Duan, Q., Leng, J., Duan, P., Hu, B. & Mao, M. An improved variable step PO and global scanning MPPT method for PV systems under partial shading condition. International Conference on Intelligent Human-Machine Systems and Cybernetics (IHMSC) 382–386 (2015).

