# Supplementary material for: Maximum Power Point Tracking for Cascaded PV-Converter Modules Using Two-Stage Particle Swarm Optimization
Source: Sci Rep. 2017 Aug 24;7:9381. doi: 10.1038/s41598-017-08009-7 (PMC5571174; doi:10.1038/s41598-017-08009-7)
Supplement: Supplementary file 1 — Supplementary Information [file 41598_2017_8009_MOESM1_ESM.doc]

**Maximum Power Point Tracking for Cascaded PV-Converter Modules Using Two-Stage Particle Swarm Optimization**

Mingxuan Mao^1,2^, Li Zhang^2^, Qichang Duan^1^,*, Hao Chen^3^

^1^Automation College, Chongqing University, Chongqing 400044, China

^2^School of Electronic and Electrical Engineering, University of Leeds, Leeds LS2 9JT, United Kingdom

^3^Judge Business School, University of Cambridge, Cambridge CB2 1AG, United Kingdom

*To whom correspondence may be addressed

E-mail: 598912836@qq.com

Phone: +44-7926285535

Fax: 01133432051

**Appendix**

**Parameters of the Fitness Function used in the proposed MPPT method:**

*I_sc_*, *I_shunt_*, and *V_out_* are expressed in (12), (13) and (14) as:

$I_{sc}=\left\{I_{scr}+k_{i}(\mathrm{T}_{\mathrm{c}}-\mathrm{T}_{r})\right\}*G$ (12)

$I_{shunt}=\frac{V_{j}}{R_{p}}\left\{1+a(1-\frac{V_{j}}{V_{br}})^{-m}\right\}$ (13)

$V_{out}=V_{j}+R_{s}I_{c}$ (14)

Diode ideality factor (*A*): 1.72;

Electron charge (*q*): 1.609×10^-19^C;

Cell absolute temperature (*T_c_*): T_c_=T+273+0.2*G;

Fixed cell series resistance (*R_s_*): 5e-5Ω;

Fixed cell parallel resistance (*R_p_*): 5e5Ω;

Temperature coefficient of the short-circuit current (*k_i_*): 1.380658e-23A;

Reference temperature (T_r_): 301.18 K°;

Short-circuit current (*I_scr_*): 3.3A;

Reverse saturation current (*I_o_*): 19.9693e-6A;

Junction breakdown voltage (*V_br_*): -4.0V;

Fraction of ohmic current (a): 0.1;

Avalanche breakdown exponent (m): 3.7.

**Figure legends：**

Figure 1. Examples of the P-V characteristics curves of a PV array composed of series-connects PV modules for the different irradiations.

Figure 2. PV system with multilevel DC- link converter and the proposed MPPT method

Figure 3. The complete flowchart of the proposed method

Figure 4. Output voltage (red line), output current (blue line) and reference voltage (green line) waveforms of five-level converter measured under the control by P&O method (column 1), the traditional PSO method (column 2) and the proposed algorithm (column 3).

Figure 5. Load power (*P_load_*), pv1 power (*P_pv1_*) and pv2 power (*P_pv2_*) of five-level converter measured under the control by P&O method (column 1), PSO method (column 2) and the proposed method (columns 3).

Figure 6. Output results of the three methods: the changing [process](javascript:void(0);) of illumination levels of two PV sources, and output power curves from the three methods.


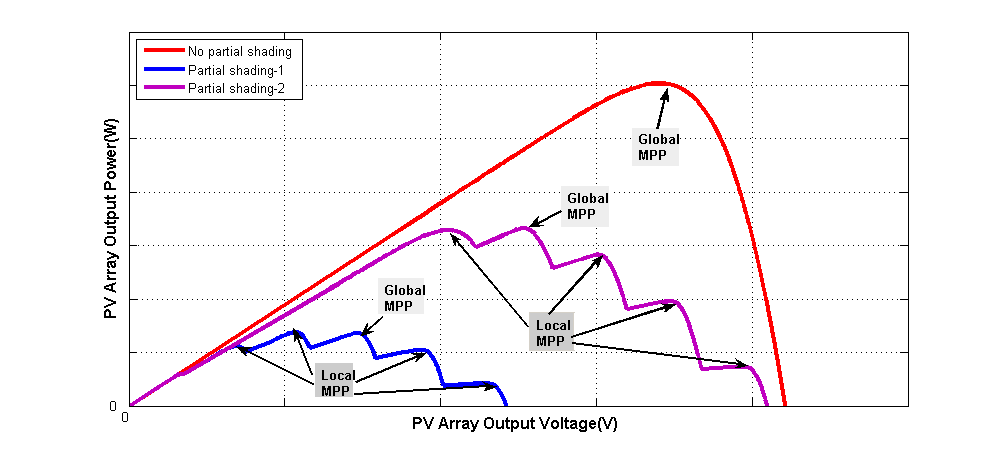


Figure 1. Examples of the P-V characteristics curves of a PV array composed of series-connects PV modules for the different irradiations.

Figure 2. PV system with multilevel DC- link converter and the proposed MPPT method

Figure 3. The complete flowchart of the proposed method

**G_1_-G_2_**

**350-500**

**500-700**

**1000-500**

**1000-700**


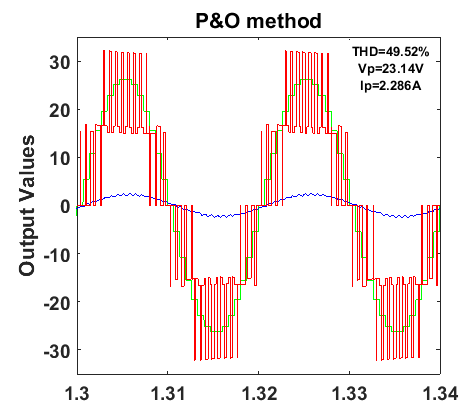

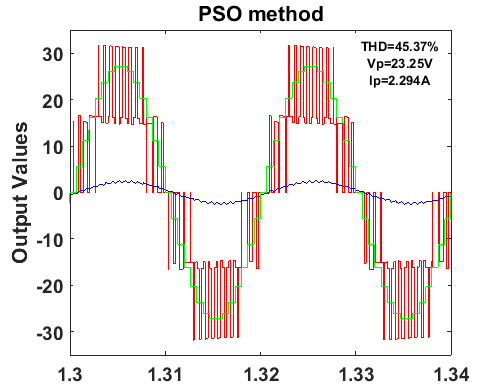

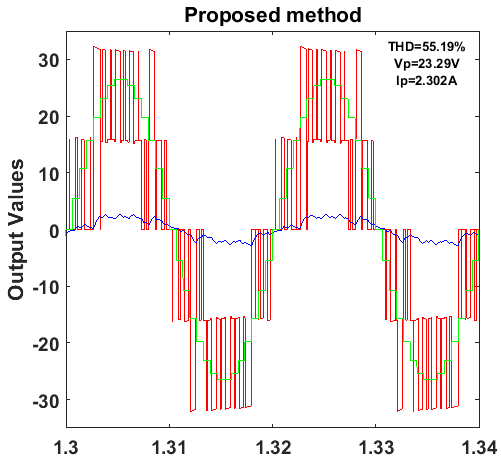


(a)


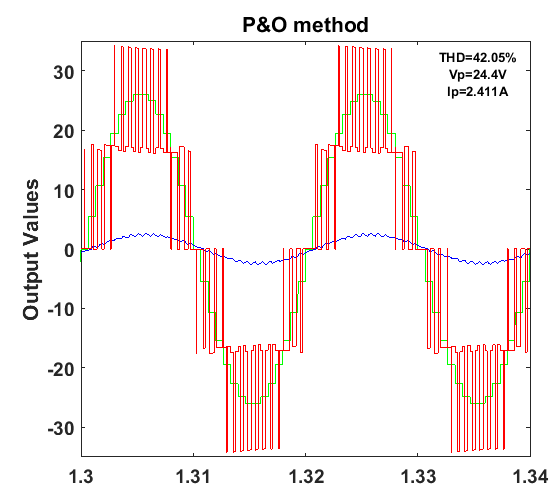

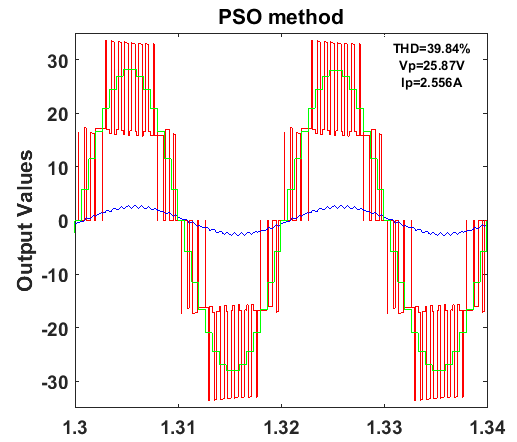

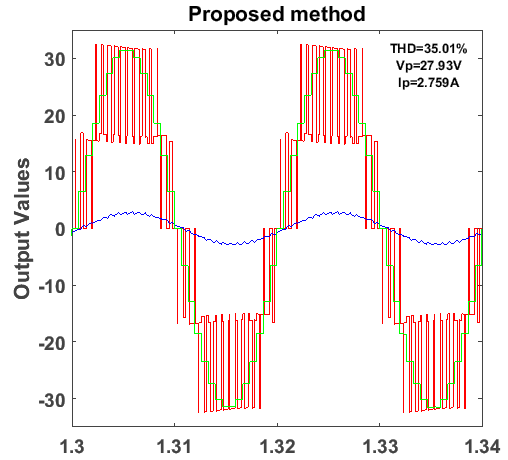


(b)


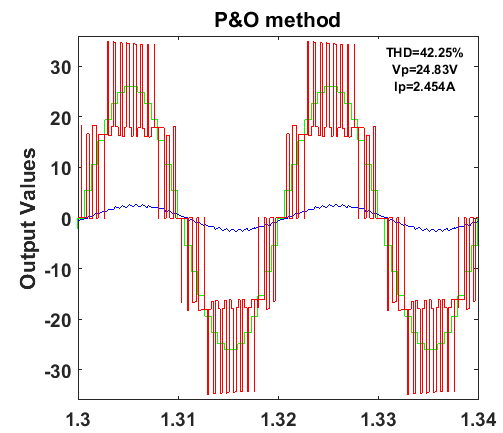

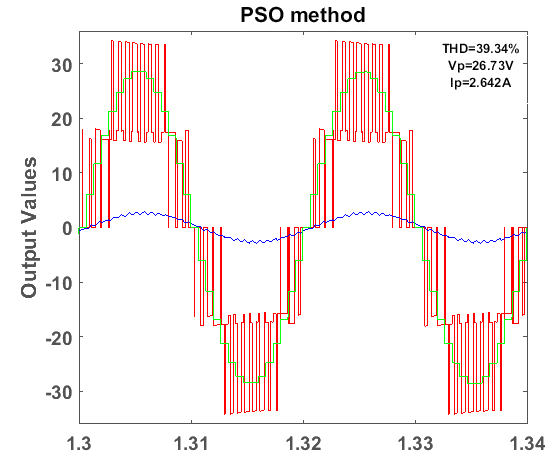

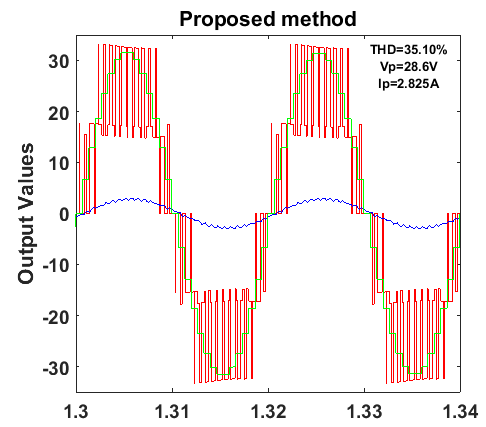


(c)


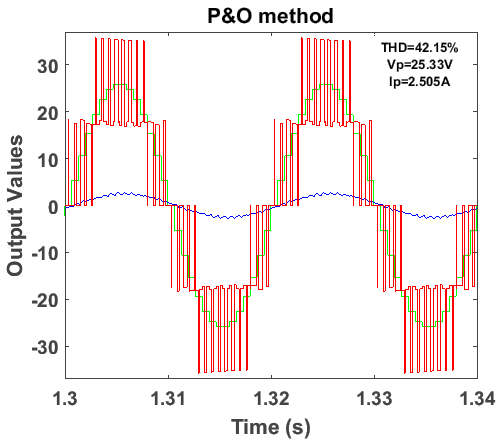

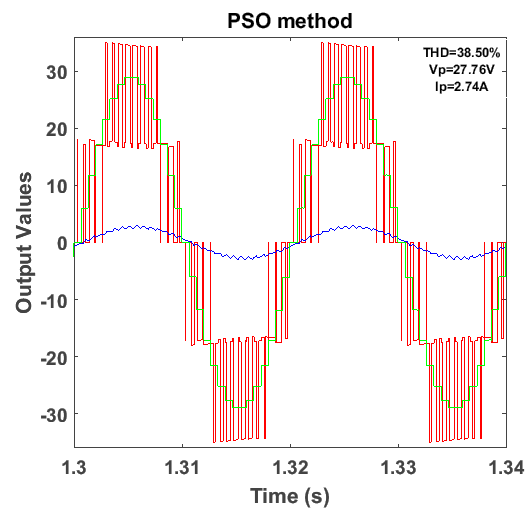

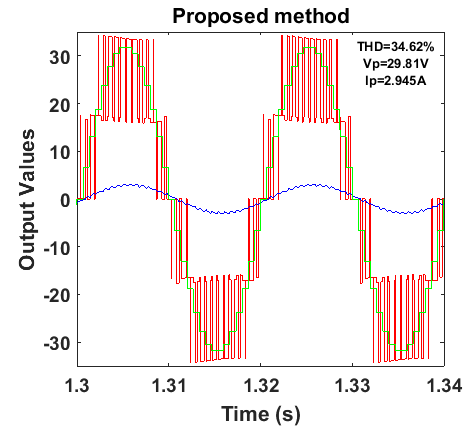


(d)

Figure 4. Output voltage (red line), output current (blue line) and reference voltage (green line) waveforms of five-level converter measured under the control by P&O method (column 1), the traditional PSO method (column 2) and the proposed algorithm (column 3).


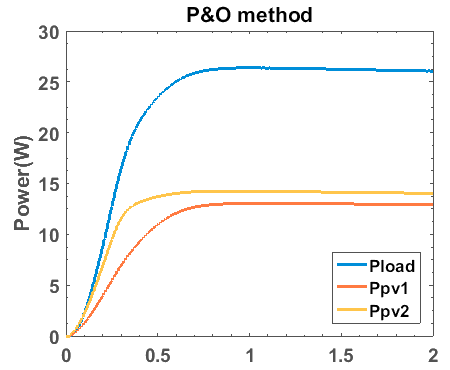

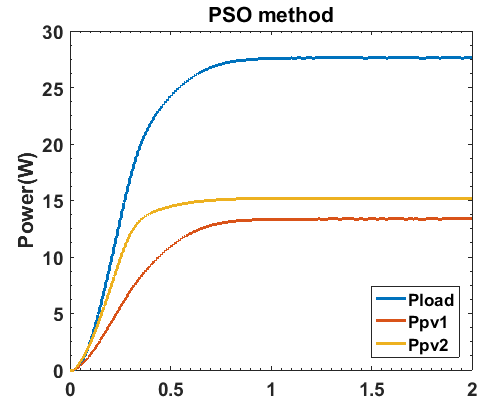

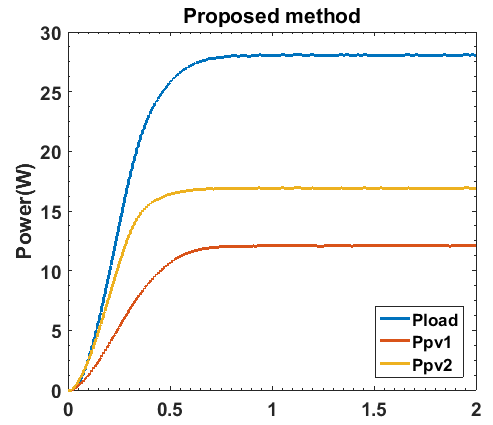


(a)


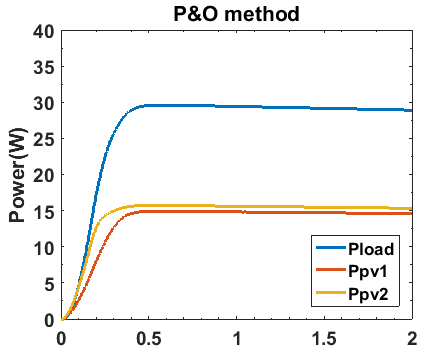

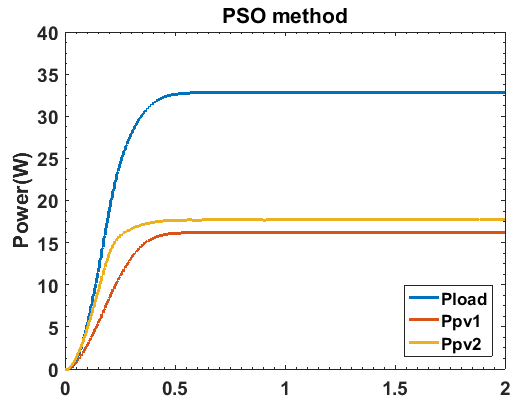

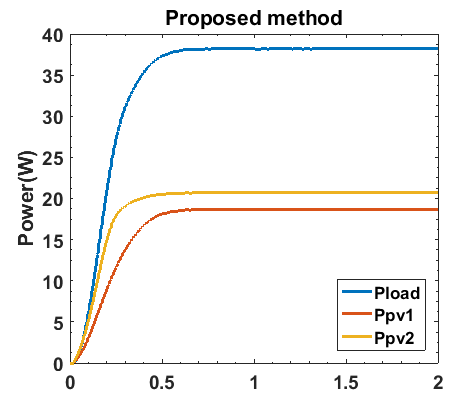


(b)


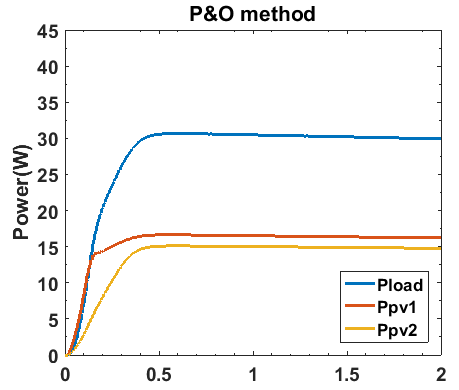

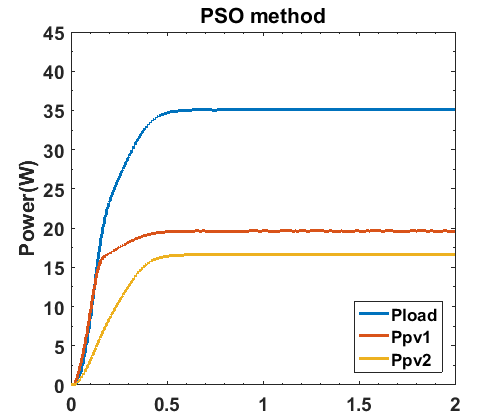

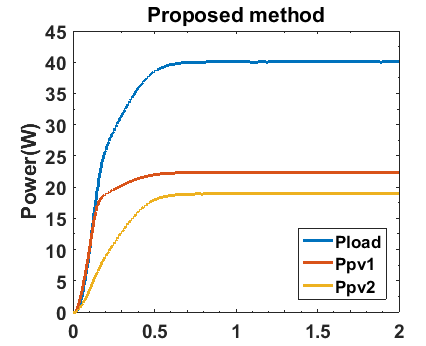


(c)


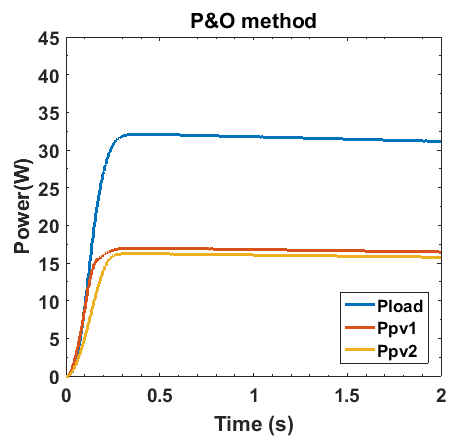

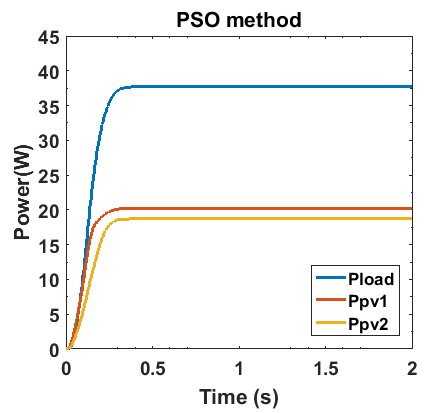

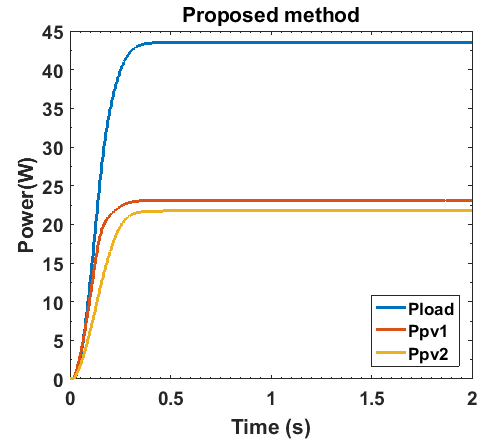


(d)

Figure 5. Load power (*P_load_*), pv1 power (*P_pv1_*) and pv2 power (*P_pv2_*) of five-level converter measured under the control by P&O method (column 1), PSO method (column 2) and the proposed method (columns 3).


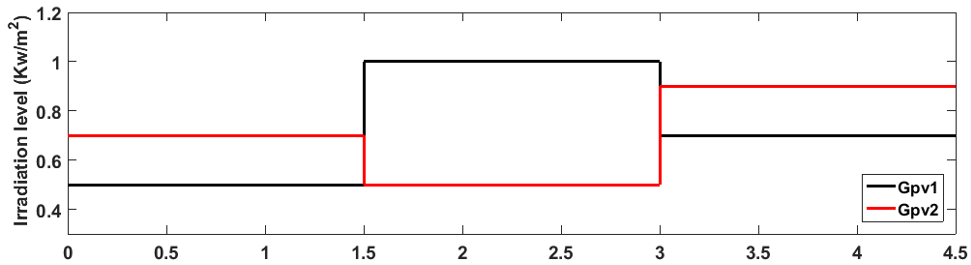


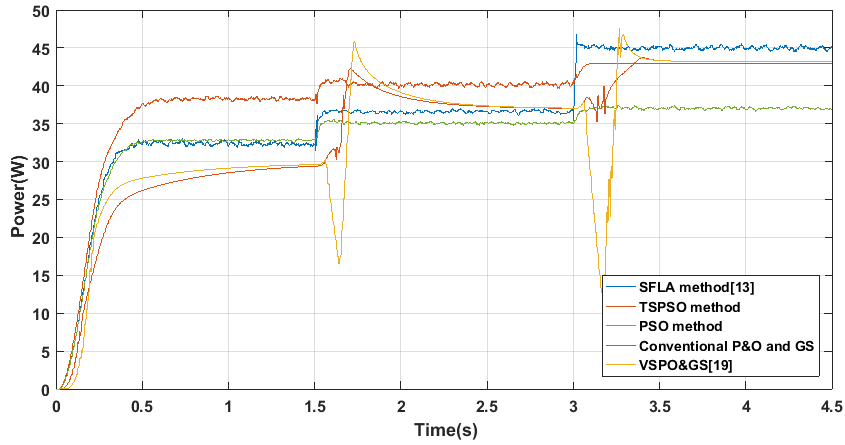


Figure 6. Output results of the three methods: the changing [process](javascript:void(0);) of illumination levels of two PV sources, and output power curves from the five methods.

**Table legends：**

Table 1. Output Generation from Two PV Sources

Table 2. Parameters of the PV panels simulated

Table 3. The basic parameters of two algorithms

Table 4. Measured power values to the load and THD values under various shading conditions

Table 1. Output Generation from Two PV Sources

| **C** | ***v*_offset1_** | ***v*_offset2_** | **Output generation per switching period *T_s_*** |
| --- | --- | --- | --- |
| **1** | **0** | **0** | Ton1  0  0  V_pv1_ |
|  | **1** | **0** |  |
|  | **0** | **1** | Ton1  V_pv1_  V_pv2_  V_pv2_ |
|  | **1** | **1** |  |
| **2** | **0** | **0** | Ton2  0  0  V_pv2_ |
|  | **0** | **1** |  |
|  | **1** | **0** | Ton2  V_pv2_  V_pv1_  V_pv1_ |
|  | **1** | **1** |  |

Table 2. Parameters of the PV panels simulated

| Symbol | Parameter | Value |
| --- | --- | --- |
| *P_mpp_* | Maximum power at 1 kW/m^2^ | 25 W |
| *V_oc_* | Open circuit voltage | 19.76 V |
| *I_sc_* | Short circuit current | 3.286 A |
| *C*_pv_ | PV source terminal capacitor | 2200 μF |
| *R* | Load resistance | 10 Ω |
| *L* | Load filter inductance | 5 mH |
| *f* | AC output frequency | 50 Hz |

Table 3. The basic parameters of two algorithms

| Method | c_1_ | c_2_ | w | S | M | J |
| --- | --- | --- | --- | --- | --- | --- |
| PSO | 0.6 | 0.8 | 0.5 | 12 | -- | 3 |
| TSPSO | 0.6 | 0.8 | self-adaption  (*ω_max_*=0.9,*ω_min_*=0.4) | 12 | 3 | 3 |

Table 4. Measured power values to the load and THD values under various shading conditions

| Case | P&O method | | | | PSO method | | | | Proposed method | | | |
| --- | --- | --- | --- | --- | --- | --- | --- | --- | --- | --- | --- | --- |
|  | P_pv1_ | P_pv2_ | P_Load_ | THD (%) | P_pv1_ | P_pv2_ | P_Load_ | THD (%) | P_pv1_ | P_pv2_ | P_Load_ | THD (%) |
| 1 | 13.02 | 14.17 | 26.29 | 49.52 | 13.36 | 15.18 | 27.62 | 45.37 | 12.09 | 16.93 | 28.08 | 55.19 |
| 2 | 14.74 | 15.51 | 29.24 | 42.05 | 16.2 | 17.70 | 32.83 | 39.84 | 18.33 | 20.69 | 36.92 | 35.01 |
| 3 | 16.41 | 14.93 | 30.29 | 42.25 | 19.62 | 16.60 | 35.09 | 39.34 | 22.34 | 18.37 | 39.17 | 35.10 |
| 4 | 16.67 | 15.96 | 31.54 | 42.15 | 20.22 | 18.72 | 37.73 | 38.50 | 23.11 | 20.73 | 42.15 | 34.62 |
